# Benchmarking single-cell hashtag oligo demultiplexing methods

**DOI:** 10.1093/nargab/lqad086

**Published:** 2023-10-11

**Authors:** George Howitt, Yuzhou Feng, Lucas Tobar, Dane Vassiliadis, Peter Hickey, Mark A Dawson, Sarath Ranganathan, Shivanthan Shanthikumar, Melanie Neeland, Jovana Maksimovic, Alicia Oshlack

**Affiliations:** Computational Biology Program, Peter MacCallum Cancer Centre, Parkville, VIC, 3010 Australia; Sir Peter MacCallum Department of Oncology, The University of Melbourne, Parkville, VIC, 3010 Australia; Computational Biology Program, Peter MacCallum Cancer Centre, Parkville, VIC, 3010 Australia; Computational Biology Program, Peter MacCallum Cancer Centre, Parkville, VIC, 3010 Australia; Sir Peter MacCallum Department of Oncology, The University of Melbourne, Parkville, VIC, 3010 Australia; Computational Biology Program, Peter MacCallum Cancer Centre, Parkville, VIC, 3010 Australia; Sir Peter MacCallum Department of Oncology, The University of Melbourne, Parkville, VIC, 3010 Australia; The Walter and Eliza Hall Institute of Medical Research, 1G Royal Parade, Parkville, VIC 3052, Australia; Department of Medical Biology, The University of Melbourne, Parkville, VIC 3010, Australia; Sir Peter MacCallum Department of Oncology, The University of Melbourne, Parkville, VIC, 3010 Australia; Centre for Cancer Research, The University of Melbourne, Parkville, VIC, Australia; Respiratory Diseases, Murdoch Children’s Research Institute, Parkville, VIC, Australia; Respiratory and Sleep Medicine, Royal Children’s Hospital, Parkville, VIC, Australia; Department of Paediatrics, The University of Melbourne, Parkville, VIC, Australia; Respiratory Diseases, Murdoch Children’s Research Institute, Parkville, VIC, Australia; Respiratory and Sleep Medicine, Royal Children’s Hospital, Parkville, VIC, Australia; Department of Paediatrics, The University of Melbourne, Parkville, VIC, Australia; Respiratory Diseases, Murdoch Children’s Research Institute, Parkville, VIC, Australia; Department of Paediatrics, The University of Melbourne, Parkville, VIC, Australia; Computational Biology Program, Peter MacCallum Cancer Centre, Parkville, VIC, 3010 Australia; Sir Peter MacCallum Department of Oncology, The University of Melbourne, Parkville, VIC, 3010 Australia; Computational Biology Program, Peter MacCallum Cancer Centre, Parkville, VIC, 3010 Australia; Sir Peter MacCallum Department of Oncology, The University of Melbourne, Parkville, VIC, 3010 Australia; School of Mathematics and Statistics, The University of Melbourne, Parkville, VIC, Australia

## Abstract

Sample multiplexing is often used to reduce cost and limit batch effects in single-cell RNA sequencing (scRNA-seq) experiments. A commonly used multiplexing technique involves tagging cells prior to pooling with a hashtag oligo (HTO) that can be sequenced along with the cells’ RNA to determine their sample of origin. Several tools have been developed to demultiplex HTO sequencing data and assign cells to samples. In this study, we critically assess the performance of seven HTO demultiplexing tools: hashedDrops, HTODemux, GMM-Demux, demuxmix, deMULTIplex, BFF (bimodal flexible fitting) and HashSolo. The comparison uses data sets where each sample has also been demultiplexed using genetic variants from the RNA, enabling comparison of HTO demultiplexing techniques against complementary data from the genetic ‘ground truth’. We find that all methods perform similarly where HTO labelling is of high quality, but methods that assume a bimodal count distribution perform poorly on lower quality data. We also suggest heuristic approaches for assessing the quality of HTO counts in an scRNA-seq experiment.

## Introduction

Improvements in droplet-based single-cell RNA sequencing (scRNA-seq) technologies have prompted growing interest in exploring variation in gene expression at cellular resolution. While costs continue to decrease, it remains expensive to separately capture and sequence individual samples. Batch effects also confound meaningful differences in gene expression between samples, and robust detection of multiplets (droplets containing two or more cells) solely from the transcriptome remains an issue (1). One solution to address these problems is to design multiplexed experiments, where samples are pooled prior to droplet capture and sequencing. The cost per sample is reduced by a factor of the number of samples sequenced, while major sample preparation batch effects within the pool are eliminated. Importantly, droplets containing cells from two or more samples can be identified. In addition, the number of cross-sample doublets can be used to estimate the expected number of within-sample doublets and thereby inform the application of other doublet detection algorithms such as scds (2) and scDblFinder (3).

Despite these advantages, it is important to carefully consider the most appropriate multiplexing protocol for the sample type(s) (4), and whether additional information is required to associate the cells with their sample of origin. For genetically distinct samples, demultiplexing can be performed based on genetic variants identified from the transcriptome using a variety of tools such as vireo and demuxlet (5,6). However, genetic demultiplexing is not possible where samples from the same individual are sequenced together (e.g. before and after treatment or different tissues from the same individual), or in model organisms, where there is typically little genetic variation between individuals. Additionally, although genetic demultiplexing is able to distinguish cells from genetically distinct individuals, it cannot provide absolute identification of the individual sample within the pool without further information about the samples, such as single-nucleotide polymorphism (SNP) genotyping.

Cell hashing is an alternative multiplexing technique. Prior to pooling, a barcoded label called a hashtag oligo (HTO) is added, one to each sample. The HTOs attach to either antibodies or lipids on the surface of the cells and the HTOs are captured and sequenced in parallel to the RNA. The antibodies bind to ubiquitous cell surface proteins (7), while the lipids incorporate into the plasma cell membrane (8).

Sequencing of the HTOs produces an HTO count matrix, an *N*_HTOs_ × *N*_droplets_ matrix consisting of the read counts for each HTO in each droplet. In an ideal scenario, each droplet contains only one cell and each cell contains only counts for the HTO corresponding to its sample of origin. In this ideal case, the demultiplexing algorithm involves simply identifying the non-empty entries in each column of the HTO count matrix. In practice, the data are noisy; droplets may contain multiple cells, HTO-conjugated antibodies/lipid molecules may not bind well to the cells or may dissociate and bind to cells from another sample in the pooling stage, or unbound HTOs may be present in droplets (7,8). Therefore, some sophistication is required for demultiplexing algorithms to distinguish the counts from the ‘true’ HTO against a background of ‘false’ counts.

In this study, we present a comparison of seven HTO demultiplexing methods: hashedDrops, HTODemux, deMULTIplex, GMM-Demux, demuxmix, BFF (bimodal flexible fitting) and HashSolo. We discuss the details of each in the ‘Materials and Methods’ section. In all cases, the fundamental goal of each method is the same: to examine the counts of each HTO in a droplet and determine the sample of origin of each cell. Conceptually, this is achieved by separating the signal from the oligo bound to the cell in the sample preparation stage (‘positive’ HTOs) from ambient counts that arise due to contamination (‘negative’ HTOs). Droplets with no positive HTOs are classified as ‘negative’ or ‘unknown’. Droplets with more than one positive HTO are classified as doublets/multiplets. Those with only one positive HTO are classified as singlets. Here, we use three data sets to assess the performance of each demultiplexing method by comparing the assignments from HTO demultiplexing to assignments from genetic demultiplexing on the same data. Recent work has shown that performance of HTOs varies between technologies and tissue types (4,9), and the data sets herein use both antibody-derived and lipid-based HTOs and incorporate liquid and solid tissue types. First, we suggest some visualizations for assessing the quality of HTO tagging. Next, we compare each method’s performance on data whose labelling quality ranges from good to poor. We find that all methods perform similarly when the labelling is of high quality. However, with lower quality labelling, methods that make simplistic, explicit assumptions about the data perform worse than those that take a more flexible approach.

## Materials and methods

### Single-cell data generation

The bronchoalveolar lavage (BAL) data set is derived from CITE-seq experiments of 24 samples of paediatric BAL. Samples were collected, cryopreserved and thawed as previously described (10). Live, single cells were sorted using a BD FACS Aria fusion and resuspended in 25 μl of cell staining buffer (BioLegend). Human TruStain FcX FC blocking reagent (BioLegend) was added according to manufacturer’s instructions for 10 min on ice. Each tube was made up to 100 μl with cell staining buffer and TotalSeq hashtag reagents (BioLegend) were added to each sample for 20 min on ice. Cells were washed with 3 ml cell staining buffer and centrifuged at 400 × *g* for 5 min at 4°C. Supernatant was discarded and each sample resuspended at 62 500 cells/100 μl following which 100 μl of each sample was pooled into one tube. Pooled cells were centrifuged at 400 × *g* for 5 min at 4°C, supernatant discarded and resuspended in 25 μl cell staining buffer and 25 μl of TotalSeq-A Human Universal Cocktail v1.0 (BioLegend) for 30 min on ice. This cocktail contains 154 immune-related surface proteins. Cells were washed in 3 ml cell staining buffer and centrifuged at 400 × *g* for 5 min at 4°C. Following two more washes, cells were resuspended in phosphate-buffered saline + 0.04% bovine serum albumin for Chromium captures. Single-cell captures, library preparation and sequencing were performed as we have described previously (11).

Data for the ovarian tumour data set were taken from (12), and were provided as a matrix of HTO counts along with the genetic assignments from vireo.

For the cell line data set, three human lung cancer cell lines, H1792, H3122 and H358, were labelled with a different 3′ lipid-modified oligo (LMO) as in (8). Cell lines were pooled in a 1:1:1 ratio and the pool was used for three separate captures with the 10x Chromium system using the 10x Genomics NextGEM 3′ Single-Cell Gene Expression Solution (10x Genomics). After single-cell capture, scRNA libraries were generated according to the manufacturer’s recommendations and LMO library preparation was performed as described previously (8). LMO count matrices were generated from fastq files using CITE-seq-count v1.4.3.

### Genetic demultiplexing

For both data sets, genetic donors were assigned to the samples by first performing SNP genotyping using cellSNP-lite (v1.2.0 for the BAL data; v1.2.1 for the cell line data) (13). We used a list of common variants from the 1000 Genomes Project (14) and filtered SNPs with <20 unique molecular identifiers or <10% minor alleles, as recommended in the cellSNP-lite manual. We then used vireoSNP 0.5.6 (5) for demultiplexing using the output of cellSNP-lite as the cell data and no additional donor information. More details are provided in (11).

### Calculating the *F*-score

For each possible HTO assignment, we calculate the true positive rate TP, which is the fraction of cells with that HTO assignment that have the corresponding vireo assignment; the false positive rate FP, which is the fraction of cells with that HTO assignment and a different genetic assignment; and the false negative rate FN, which is the fraction of cells with the corresponding genetic assignment but a different HTO assignment. Our key metric is the *F*-score, which is defined as


\begin{eqnarray*} F = \frac{{\rm TP}}{{\rm TP} + 1/2 ( {\rm FP} + {\rm FN})}. \end{eqnarray*}

*F* is the harmonic mean of the precision and recall, and can vary between 0 and 1, with a higher *F*-score implying better performance.

### Overview of demultiplexing methods in this comparison

#### hashedDrops

hashedDrops, part of the DropletUtils package (15), is a simple threshold-based classifier. First, the HTO count matrix is corrected for the ambient counts of each HTO in the data (either before or after filtering out empty droplets). It then ranks the HTO counts in each droplet. Assignments are determined solely by the log-fold change (LFC) between the highest and second highest counts in a droplet, relative to the median counts for that HTO. First, doublets are called where the LFC of the second highest HTO is greater than a user-defined number of median absolute deviations (MADs) above the median and also greater than another user-defined threshold. If a droplet is not assigned as a doublet, singlet assignments are determined by checking that the LFC of the HTO with the highest count in each droplet is greater than a user-defined threshold and is also not less than a user-defined number of MADs below the median. While less sophisticated than other demultiplexing methods, hashedDrops has the advantage of making very few assumptions about the data, and is easily configurable by the user. However, as the results are very sensitive to the choice of the hard thresholds, their values should be carefully considered. We explore the effect of varying the singlet threshold parameter in Supplementary Figure S3.

#### HTODemux

HTODemux (7), included in the Seurat package, uses a clustering-based approach. The HTO counts are normalized using the centred log ratio (CLR) transformation. Then, an unsupervised *k*-medoids clustering is performed, with *k* = *N*_HTOs_ + 1. For each HTO, cells are identified as positive or negative in a two-step procedure. First, the cluster with the lowest expression count for each HTO is defined as the ‘negative’ cluster, and a negative binomial distribution is fitted to the counts in that cluster. For the droplets outside that cluster, droplets with HTO counts above a user-defined quantile (0.99 by default) are assigned as positive for the HTO. After performing this procedure on all HTOs, droplets that have been assigned positive for more than one HTO are classified as multiplets, droplets with no positive assignments are classified as negative and the droplets assigned positive for only one HTO are classified as singlets. We explore the effect of varying the quantile threshold in Supplementary Figure S4.

#### GMM-Demux

Like HTODemux, GMM-Demux (16) uses the CLR-transformed HTO counts. In well-behaved data, the distribution of the CLR-transformed counts of each HTO is bimodal, with the lower peak corresponding to the ‘negative’ background and the higher peak corresponding to the true ‘positive’ counts. GMM-Demux fits a two-component Gaussian mixture model to the distribution of each HTO, and uses Bayesian estimation to assign each droplet to the higher or lower peaked distribution for each HTO. Droplets with only one positive HTO assignment are classified as singlets and droplets with no positive assignments are classified as negative, while droplets with multiple positive assignments are classified as multiplets, with the identity of the most probable HTOs in each multiplet included in the output. Every positive assignment is given a confidence score between 0 and 1, and a user-defined confidence threshold (0.8 by default) can be adjusted to be more or less strict with the output classifications. We explore the effect of varying the confidence threshold set in Supplementary Figure S5.

#### demuxmix

demuxmix (17) is similar to GMM-Demux but uses a negative binomial mixture model on the untransformed HTO counts, rather than a mixed Gaussian on the CLR-transformed counts. For each HTO, all cells are clustered into positive and negative clusters using *k*-means clustering. Cells with very high counts are marked as outliers, and the non-outliers are fitted to a two-component negative binomial distribution using an expectation–maximization algorithm. demuxmix can also leverage the RNA counts to improve performance, using the number of detected genes in the RNA library as a covariate in the mixture model. Using this additional RNA information with the BAL data set showed no significant improvement on either data set in this paper, so the results presented are based on the HTO counts only.

#### deMULTIplex

deMULTIplex (8) uses an iterative approach. First, a kernel density estimator is used to smooth the log-normalized HTO counts. For each HTO, an initial threshold for positive classification is defined as the highest maximum (assuming a bimodal normalized count distribution), while the initial threshold for negative classification is the mode. Then, the algorithm sweeps through the quantile range between these two thresholds to find the value that classifies the largest proportion of the data as singlets. Each droplet is then compared against each HTO-specific threshold, being classified as negative, singlet or multiplet based on the number of HTOs for which it passes. All negatively classified droplets are removed from the count matrix, and the process is repeated until successive iterations identify no additional negative droplets. While the thresholds for singlets and doublets can be adjusted manually, the default option searches for the value that maximizes the fraction of singlet assignments, and our results use this automatic threshold-determining mode.

#### BFF

BFF (18) also assumes a bimodal count distribution. It operates in two modes, BFF_raw_ and BFF_cluster_. The first mode, BFF_raw_, smooths the count distribution using a kernel density estimator, much like deMULTIplex. The threshold between positive and negative classifications in this case is the local minimum between the two peaks. The second mode, BFF_cluster_, is similar, but includes an additional layer of normalization, called bimodal quantile normalization, before finalizing classifications. The level of smoothing on the counts can be selected by the user; however, our results are based on the default, which searches for an optimal value.

#### HashSolo

HashSolo (19) is a Bayesian method that models the overall count distribution across all cells as a mixture of two log-normal distributions corresponding to signal and noise. For each cell, it looks at the two highest counts and computes the likelihood of both belonging to the noise distribution (negative), one belonging to the signal and one belonging to the noise (singlet), or both belonging to the signal (doublet). It then returns the assignment with the highest Bayesian evidence. The prior is the fraction of singlets, doublets and negative cells within the sample. Based on the vireo results, we use a prior of 75% singlets, 20% doublets and 5% negatives. We also run HashSolo with a negative fraction prior between 1% and 10% and a doublet fraction prior between 10% and 30%. This has a negligible effect on the posterior assignments, with the average *F*-score varying by <1% in all batches.

## Results

### Evaluation data sets

We perform our comparison of hashtag demultiplexing methods on six tagging experiments across three data sets, each using different tagging technologies. The first data set, the BAL data set, contains 24 genetically distinct samples of BAL fluid tagged with TotalSeq-A antibody-derived tags (ADTs) (7). These samples were processed in three batches of eight pooled samples, each with two captures per batch. Batch 1 contains 24 091 droplets, batch 2 contains 48 841 droplets and batch 3 contains 62 306 droplets.

The second data set, the ovarian tumour (OT) data set, contains eight genetically distinct samples of high-grade serous ovarian tumours, tagged with TotalSeq-B ADTs. These samples were processed in two batches of four samples each; batch 1 contains 12 510 droplets and batch 2 contains 9547 droplets (12).

The third data set, the cell line (CL) data set, contains samples from three human lung cancer cell lines, which are tagged with MULTI-seq LMOs (8). The cell line data set contains 45 977 droplets.

For each data set, vireo (5) is used to assign cells to individuals with default settings (see the ‘Materials and Methods’ section) and these are used as the ‘ground truth’ to assess the accuracy of the HTO demultiplexing methods. Tests of vireo on simulated data sets show close to 100% accuracy on singlets and >90% accuracy on between-sample doublets (1). While such performance may be optimistic for real-world data sets, vireo returns similar assignment scores for all three batches in the BAL data set (Supplementary Figure S2). This consistent performance is in contrast to the HTO demultiplexing methods.

Throughout this paper, we make a distinction between the labels assigned to cells using vireo and the labels of the corresponding HTOs. The vireo labels are denoted by the data set name followed by an alphabetical suffix, e.g. BAL A, CL B and OT C, while the corresponding HTO labels contain a numeric suffix, e.g. BAL 1, CL 2 and OT 3.

### QC visualization

**Figure 1.**
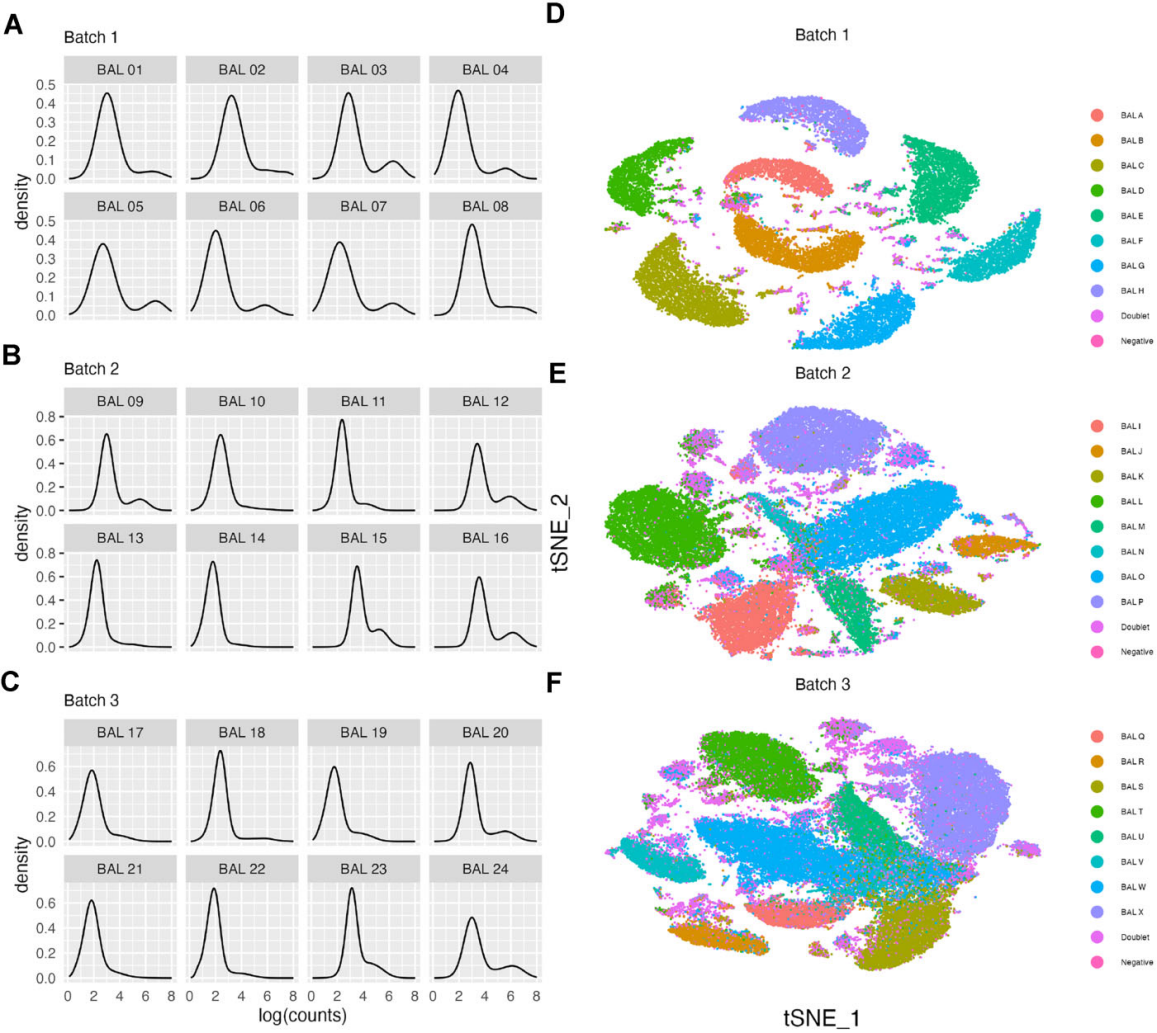
Quality assessment visualizations of the BAL data set. (**A**–**C**) Probability density function of the logarithm of HTO counts for each hashtag and (**D**–**F**) tSNE dimensional reduction of HTO counts, coloured by genetic donor in batches 1 (D), 2 (E) and 3 (F).

To assess the quality of the HTO labelling and sequencing, we suggest using some common qualitative visualizations that can guide overall expectations of the performance of HTO deconvolution. Figure 1 shows the probability density function, approximated using kernel density estimation, of the logarithm of counts per cell of each HTO across the three batches in the BAL data set (Figure 1A–C). The tSNE (t-distributed stochastic neighbour embedding) dimensional reductions of the principal component analysis (PCA) of log-normalized HTO counts in each batch are also shown (Figure 1D–F). Each HTO in batch 1 of the BAL data set follows a bimodal distribution (Figure 1A), with a lower peak corresponding to the background counts in the majority of droplets and a higher peak corresponding to the cells from the tagged sample. In batches 2 (Figure 1B) and 3 (Figure 1C), some HTOs (e.g. BAL J and BAL N in batch 2, BAL U in batch 3) appear unimodal, indicating lower quality labelling. In the right column, the tSNE of batch 1 (Figure 1D) has eight distinct clusters, corresponding to the eight individual samples, with a constellation of smaller, interspersed clusters that correspond to doublets and unassigned droplets based on the genetic assignments. Batches 2 (Figure 1E) and 3 (Figure 1F) also show eight clusters; however, the boundaries of these clusters are closer than in batch 1, and overlap for some samples in batch 3. While not quantitative, the tSNE plots in Figure 1 indicate that the cells in batch 1 are well labelled, while those in batches 2 and 3 are labelled more poorly, highlighting that demultiplexing these batches is likely to be more challenging and demultiplexing less accurate (as shown in the following section). In addition, specific samples within a batch are labelled more poorly than others as indicated by the density plots of the individual HTOs and the overlapping tSNE clusters. Overall, the density and tSNE plots of the HTO counts can be used to quickly evaluate the quality of the HTO labelling. High-quality data are indicated by bimodal density plots and tSNE plots with distinct, major clusters corresponding to the number of samples.

### Quantitative comparisons of demultiplexing methods

Each of the three batches in our BAL data set contains cells from eight samples, from genetically distinct donors. Each demultiplexing method (including the genetic demultiplexing) can return one of 10 assignments for a cell: singlet, corresponding to one of the eight unique samples; doublet; or negative. We compare seven HTO demultiplexing methods: BFF (18), deMULTIplex (8), demuxmix (17), GMM-Demux (16), hashedDrops (15), HTODemux (7) and HashSolo (19). BFF has two modes, BFF_raw_ and BFF_cluster_, and we present the output of both. All of the methods we consider have some adjustable parameters that affect output; however, in our exploration, changing the default options does not significantly change the assignments. We discuss the details of each method and their parameters further in the ‘Materials and Methods’ section. The exception is hashedDrops, which uses a simple count threshold to distinguish negatives and singlets. We find that in many cases the default value of this threshold is too high, and performance (defined here as the *F*-score; see the ‘Materials and Methods’ section) is improved by lowering its value. To illustrate this, we present the hashedDrops classifications with both the default value (confident.min = 2) and the value we find maximizes the *F*-score (confident.min = 0.5). As each batch was processed across two captures, we run the demultiplexing methods on HTO data from each individual capture. However, for simplicity, the results are presented per batch as we do not observe significant variation between captures within a batch.

**Figure 2.**
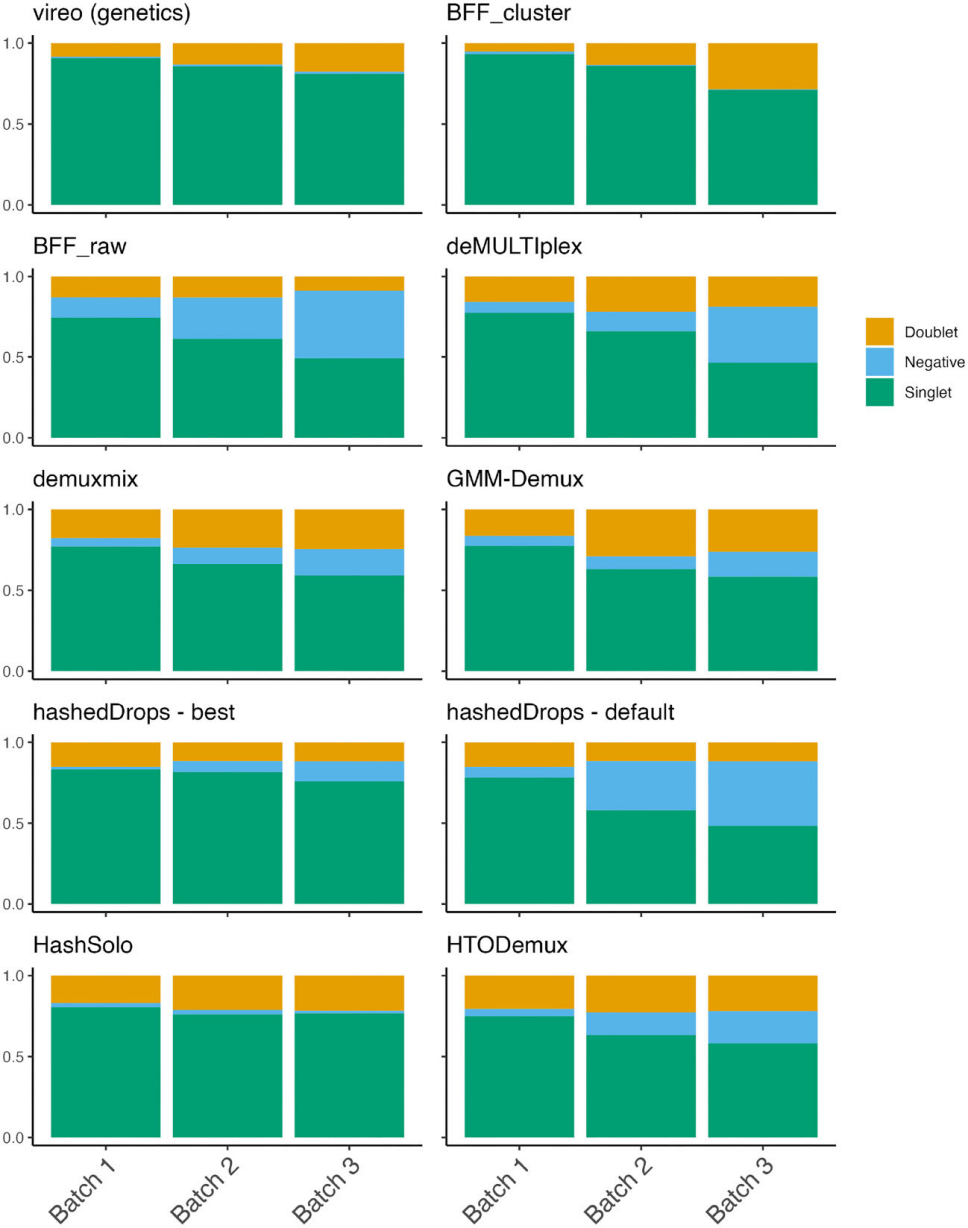
The proportion of cell assignments to singlets, doublets or negative droplets for each demultiplexing method of the BAL data set. Each panel is a method and each bar is a batch.

In Figure 2, we show the fraction of assignments in each broad category: singlet, doublet or negative, from vireo and each hashtag demultiplexing method for the three batches in the BAL data set. Two clear trends are apparent in Figure 2. First, vireo is able to assign more droplets as singlets than any of the hashtag demultiplexing methods. Second, the hierarchy of HTO tagging quality between the batches suggested by Figure 1 is confirmed in Figure 2. The fraction of negative droplets increases from batch 1 to batch 3 for most methods. The exception to both is BFF_cluster_, which assigns slightly more singlets than vireo in batches 1 and 2, and assigns fewer negative droplets in batches 2 and 3 than in batch 1. HashSolo assigns fewer negative cells than any other method, excluding vireo and BFF_cluster_, and assigns a similar fraction of doublets in each batch, with the doublet fraction greater than vireo in all batches.

**Figure 3.**
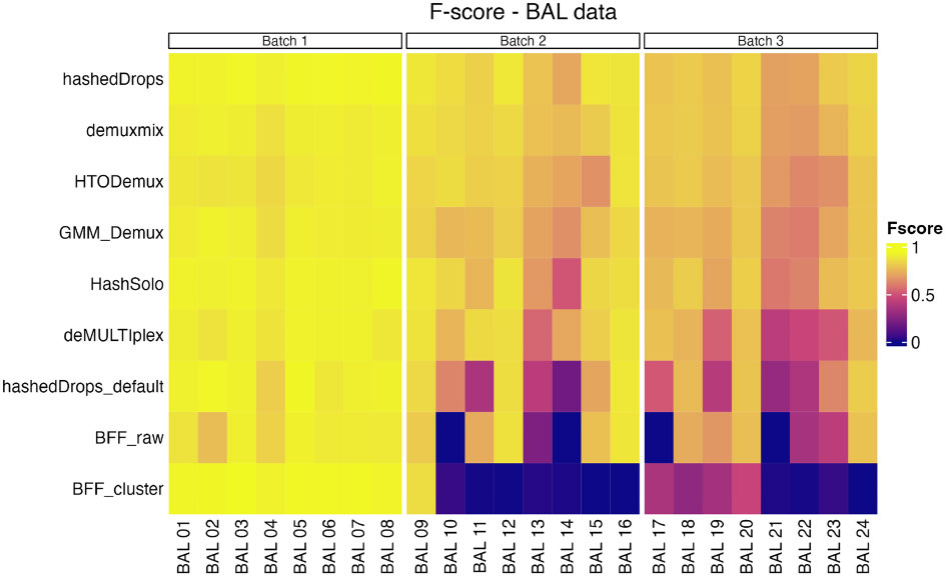
*F*-scores of each singlet assignment from each demultiplexing method for the BAL data set. Left panel: batch 1; middle panel: batch 2; right panel: batch 3. Methods are ordered from highest to lowest average *F*-score across all three batches.

We next compare the specific individuals allocated by the singlet assignments of each HTO demultiplexing method to the ‘ground truth’ of genetic assignments from vireo. To quantitatively assess their performance, we calculate the *F*-score (see the ‘Materials and Methods’ section), a statistic that is the harmonic mean of precision and recall. The *F*-score ranges between 0 and 1, with a higher value indicating better performance. Figure 3 shows a heatmap of the *F*-score of each method, for each possible singlet assignment, split by batch.

Table 1 shows the mean *F*-score of each sample for each method in all data sets. The BAL data are shown in the second, third and fourth columns. Numerical values of the *F*-scores for all samples and methods are included in the Supplementary Data. Figure 3 and Table 1 show that all methods perform well for batch 1. The overall performance of all methods drops for batches 2 and 3 and some methods begin to show significant performance differences between batches. Notably, BFF_cluster_, which has the highest mean *F*-score in batch 1, has *F* < 0.1 for all HTOs except BAL 9 in batch 2 and all HTOs except BAL 17, BAL 18, BAL 19 and BAL 20 in batch 3. BFF_raw_ is unable to classify any cells to BAL 10 or BAL 14 in batch 2 or BAL 17, BAL 21 and HTO 14 in batch 3. hashedDrops has higher scores in all batches with optimized parameters than with the default settings, and has the highest mean *F*-score of all methods in batches 2 and 3. Demuxmix, GMM-Demux, HashSolo and HTODemux show consistent performance across all three batches.

**Table 1.** Singlet fraction and mean *F*-score of each demultiplexing method for all batches in all data sets

Metric	BAL batch 1	BAL batch 2	BAL batch 3	OT batch 1	OT batch 2	CL
Genetic singlet fraction	21 851/24 091	41 816/48 841	50 496/62 306	11 296/12 510	8683/9547	40 118/45 977
*F*_mean_ (hashedDrops)	0.930	**0.847**	**0.785**	**0.679**	**0.813**	0.864
*F*_mean_ (demuxmix)	0.907	0.833	0.775	0.511	0.506	0.743
*F*_mean_ (HTODemux)	0.892	0.794	0.742	0.620	0.654	0.737
*F*_mean_ (GMM-Demux)	0.905	0.770	0.720	0.645	0.632	0.823
*F*_mean_ (HashSolo)	0.920	0.783	0.737	0.606	0.688	0.806
*F*_mean_ (deMULTIplex)	0.907	0.791	0.624	0.218	0.684	0.828
*F*_mean_ (hashedDrops—default)	0.907	0.613	0.573	0.406	0.46	0.663
*F*_mean_ (BFF_raw_)	0.879	0.539	0.466	0	0.623	0.791
*F*_mean_ (BFF_cluster_)	**0.935**	0.119	0.188	0	0.408	**0.874**

The best-performing method for each batch is indicated in bold.

Next, we investigate doublets in more detail. As shown in Figure 2, almost all methods assign more doublets than vireo. Assigning true doublets as singlets is potentially a more significant source of error in downstream analysis, such as cell type identification, than misclassifying a true singlet as a doublet, negative or incorrect singlet (20). Therefore, we exclude the doublet and negative classifications from our *F*-score analysis above. Instead, we perform a separate, complementary analysis of how each HTO demultiplexing method classifies the genetic doublets. For each batch in the BAL data set, we take the droplets assigned as doublets by vireo, and look at which broad category (i.e. doublet, singlet or negative) they are assigned by each of the HTO demultiplexing methods. For this analysis, the best-performing method is the one that minimizes the number of ‘true’ doublets assigned as singlets. Since both negative droplets and doublets are typically excluded from downstream analysis, misclassification of genetic doublets as negatives is relatively unimportant.

**Figure 4.**
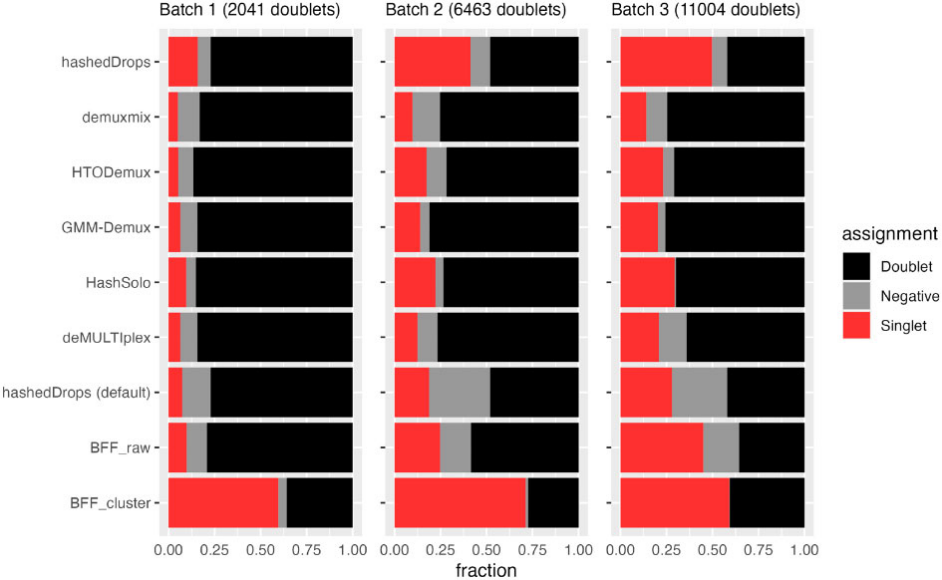
Fraction of genetic doublets assigned by each HTO demultiplexing method to doublets (black), negatives (grey) or singlets (red) in the BAL data set. Methods are in the same order as Figure 3.

Figure 4 shows the fraction of vireo doublets assigned by each method as doublet, singlet or negative. Figure 4 illustrates several points not apparent in Figure 3 and Table 1. First, BFF_cluster_, which has the highest *F*-score for batch 1, has the worst performance on the doublets, assigning more than half of the genetic doublets in that batch as singlets. Second, while adjusting the parameters of hashedDrops from their default values improves the *F*-score, the number of incorrectly assigned genetic doublets approximately doubles in all batches. Third, the other best-performing methods based on *F*-score, demuxmix, HTODemux and GMM-demux, perform well on the doublet analysis as well, assigning <20% of genetic doublets as singlets in all batches, though HashSolo performs slightly worse, with ≈30% of genetic doublets identified as singlets.

## Ovarian tumour data

The second data set we analyse consists of eight samples from high-grade serous ovarian carcinoma patients from (12). Unlike the BAL data samples, which are liquid, these tumour samples require dissociation prior to hashtagging and single-cell sequencing. The samples were tagged with TotalSeq-B ADTs and processed in two batches of four samples each, with 12 510 droplets in batch 1 and 9547 droplets in batch 2.

Genetic demultiplexing with vireo positively assigns >90% of droplets as singlets for both batches (12). Initial quality control (QC) of the HTO counts (Supplementary Figure S6) suggests poor performance of the hashtagging in this experiment, which is borne out by the low *F*-scores of all demultiplexing methods on this data set.

**Figure 5.**
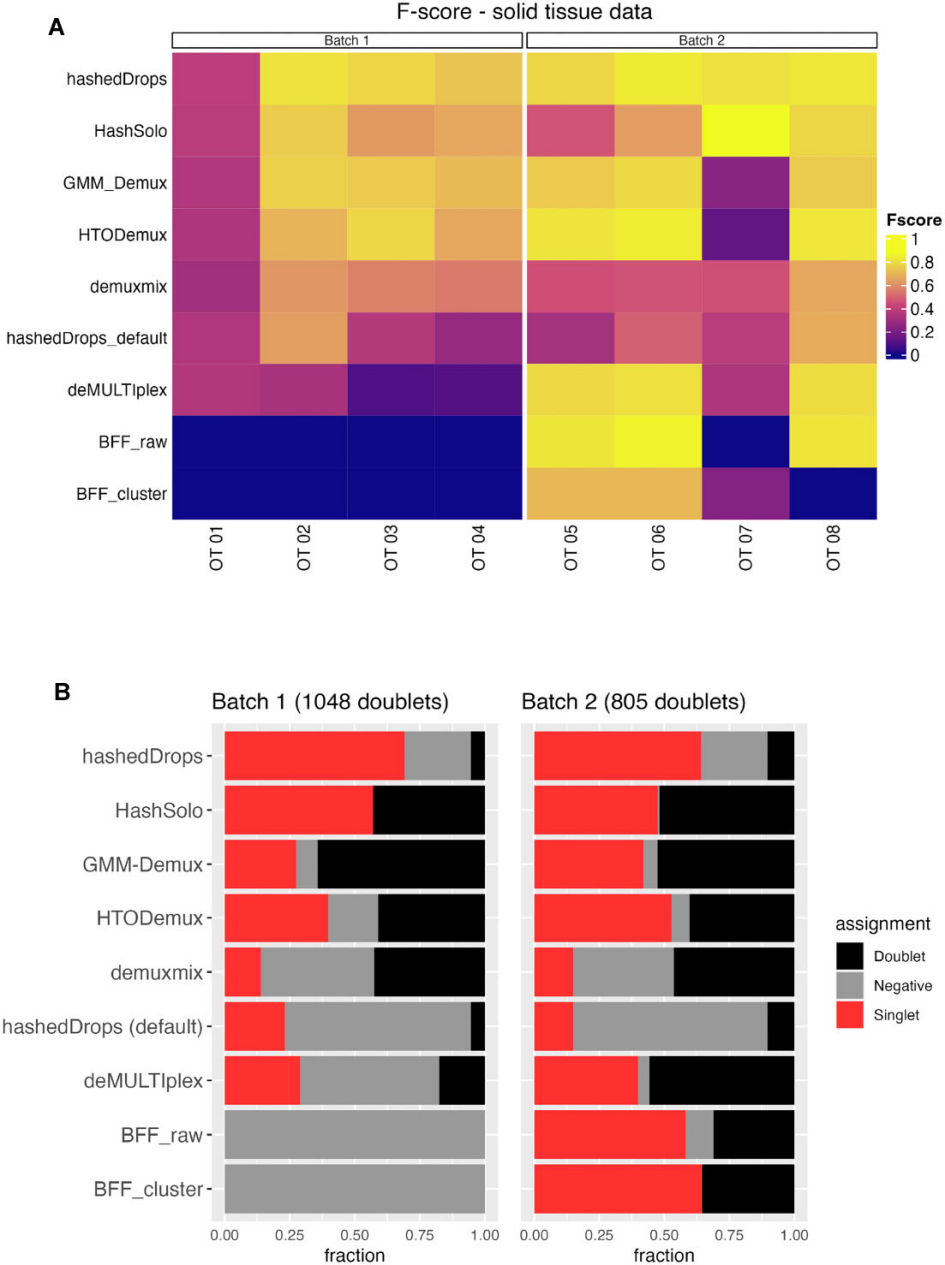
(**A**) Heatmap of *F*-scores for each demultiplexing method on each sample from the ovarian tumour data set. (**B**) Fraction of the genetic doublets in each batch assigned to different categories by each method. Methods are ordered from highest to lowest average *F*-score across the two batches.

Figure 5 shows a heatmap of the *F*-scores for each method on each sample and the assignment of genetic doublets as doublets, singlets or negatives by each method. The mean *F*-scores for each method in each batch are shown in the fifth and sixth columns of Table 1. In general, the performance of all demultiplexing methods is worse for both batches in the ovarian tumour data set than the BAL data. This is possibly due to the additional dissociation step in the sample processing (4). As for the BAL data set, using hashedDrops is a trade-off between *F*-score and doublet classification accuracy. HashSolo, GMM-Demux and HTODemux all perform relatively well, while demuxmix, the method with best overall performance on the BAL data set, falls behind on *F*-score but makes fewer errors on doublets. How these two factors should be weighed against one another cannot be answered objectively for all cases, especially as the overall fraction of doublets depends on the overall number of cells per batch (1). For example, in batch 3 of the BAL data set, using hashedDrops instead of demuxmix improves the mean *F*-score by only 0.01, while an additional ≈3000 doublets are incorrectly labelled as singlets. In batch 1 of the ovarian tumour data set, however, using hashedDrops instead of demuxmix improves the mean *F*-score by 0.307, while misclassifying ≈500 doublets as singlets. In the former case, demuxmix is clearly superior, while in the latter case it might make sense to prefer hashedDrops. BFF_raw_ and BFF_cluster_ again perform poorly, especially on batch 1, where both fail to classify any cells.

## Cell line data

We perform the same analysis on a third data set, the cell line data set, consisting of three samples from genetically distinct human lung cancer cell lines. Here, H3122, H358 and H1792 cells were tagged with different MULTI-seq LMOs (8), a different tagging technology to the ADTs used on the BAL and ovarian tumour samples. These samples were pooled together and processed in one batch across three captures, with 45 977 total droplets.

**Figure 6.**
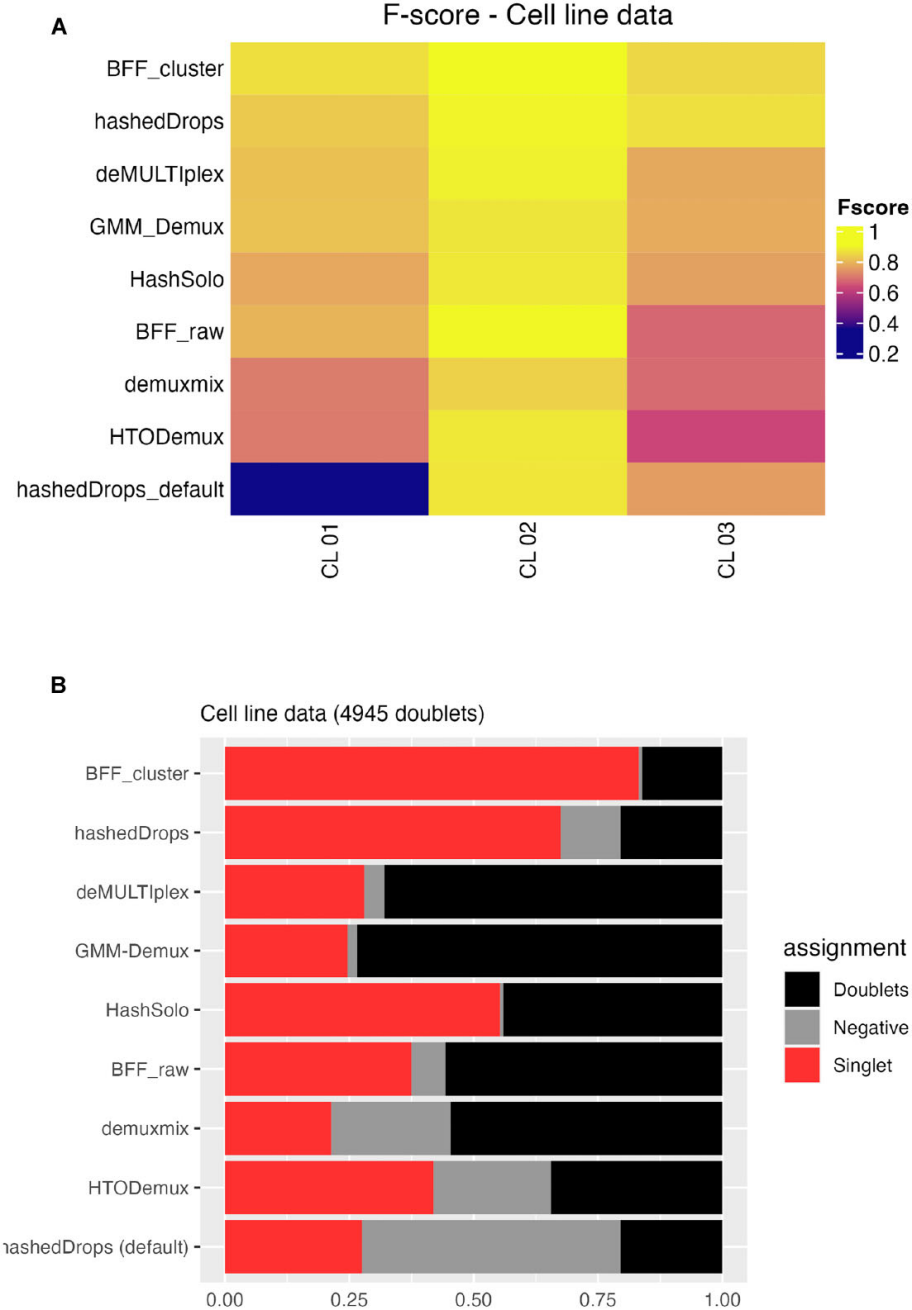
(**A**) Heatmap of *F*-scores for each demultiplexing method on each sample from the cell line data set. (**B**) Fraction of the genetic doublets assigned to different categories by each method. Methods are ordered from highest to lowest average *F*-score.

Since both the ADT and LMO technologies produce an *N*_HTOs_ × *N*_droplets_ count matrix with similar distributions (see Supplementary Figure S7), we expect the demultiplexing methods to perform similarly on the LMO and ADT data. Figure 6 shows the *F*-score for each method on each of the three samples in this data set (Figure 6A), as well as the categorical assignments of the 4945 genetic doublets (Figure 6B). Figure 6 and Supplementary Figure S7 show that the cell line data are somewhere between the quality of batches 1 and 2 of the BAL data, and the performance of each of the methods is similar. Based on *F*-score alone (Figure 5A), BFF_cluster_ performs best; however, looking at Figure 5B, we see that >75% of genetic doublets are assigned as singlets. Based on the two metrics, we find that deMULTIplex, GMM-Demux and demuxmix perform well, hashedDrops with default parameters and HTODemux perform relatively poorly, and hashedDrops with lowered thresholds and HashSolo perform well based on the *F*-score, but misidentify nearly 60% of genetic doublets as singlets—more than twice as many as the best-performing methods.

## Discussion

As sample multiplexing becomes more common in scRNA-seq experiments, reliable demultiplexing of cells becomes paramount. We benchmark seven methods for cell demultiplexing based on HTO data. Of the methods we consider, demuxmix shows the best overall performance across the three data sets included in this study, using our two criteria of accurately classifying singlets and rarely misclassifying genetic doublets as singlets. However, the difference between demuxmix, GMM-Demux and HTODemux is small, and all should perform relatively well on most data sets. Furthermore, for data sets with lower quality hashing, we suggest that it may be prudent to trial several of these methods to maximize the number of positively identified singlets. Looking only at the *F*-score, hashedDrops is the best overall performer when the threshold for confident detection is lowered. However, this comes at the cost of misclassifying doublets as singlets. deMULTIplex and BFF methods perform especially poorly on lower quality hashing data, as they rely on measurement of two peaks in a density estimate of the transformed counts, which may not exist when a large number of background counts are present.

Our results are broadly consistent for hashtagging using ADTs and LMOs, as well as liquid and solid tissues, indicating that the performance of the demultiplexing methods is agnostic to the choice of tagging protocol.

Although most of the tools are straightforward to run, and interact well with popular single-cell analysis packages, there are some important usability differences. demuxmix is part of the Bioconductor ecosystem and can easily be run in R. As it only requires an HTO count matrix to return assignments, it can be incorporated as part of a Bioconductor- or Seurat-based single-cell analysis pipeline. HTODemux is part of the Seurat package and requires a Seurat object as input, and therefore runs most easily alongside other Seurat tools for single-cell analysis. HashSolo is part of the scanpy ecosystem and can be run easily as part of a scanpy analysis pipeline. However, although HashSolo performs well on high-quality data, its tendency to misidentify genetic doublets as singlets means that care should be taken on superloaded data. GMM-Demux is a command-line tool, which may provide a barrier to entry for some users, although wrappers such as cellhashR (18) can be used to run it from R.

We demonstrate two simple visualization methods to assess the quality of hashtag data, and confirm that if the probability density of counts follows a bimodal distribution, and the counts separate into well-defined clusters on a dimensional reduction plot, then all demultiplexing methods perform well. However, if these conditions are not met, demultiplexing algorithms that explicitly assume bimodal distributions (such as deMULTIplex and BFF) fail to correctly assign some droplets to their samples of origin. Threshold-based methods, such as hashedDrops, can perform well but make a trade-off between greater recovery of singlets and false positives. More sophisticated methods, such as the clustering-based HTODemux and demuxmix, and the Bayesian estimation-based methods GMM-Demux and HashSolo perform best and most consistently on both high- and low-quality hashtag data.

Low-quality hashtag data do not imply low-quality RNA expression data; importantly, the two are largely uncorrelated (see Supplementary Figure S1). We show that the difference between demultiplexing methods becomes more pronounced as the quality of the hashtag data reduces. Therefore, maximizing performance of demultiplexing methods on lower quality hashtag data is particularly important to prevent otherwise good quality cells being excluded in a single-cell analysis.

For samples with similar or identical genetic backgrounds, labelling of cells before pooling will continue to be an important strategy and this manuscript provides a benchmark for applying these methods. While genetic demultiplexing has been shown to be accurate for deconvolving samples from outbred populations (1), there is potential for information from the genetics and hashtags to be used together for more accurate deconvolution (21,22) and we expect to see continued developments in this area.

## Supplementary Material

lqad086_Supplemental_Files

## Data Availability

Code to reproduce the analysis in this paper can be viewed at https://oshlacklab.com/hashtag-demux-paper, and the raw count data and vireo assignments for the BAL and cell line data sets can be accessed through Zenodo at https://zenodo.org/record/8304003 (DOI: 10.5281/zenodo.8304002). The ovarian tumour data set is taken from the preprint ‘Performance of computational algorithms to deconvolve heterogeneous bulk tumour tissue depends on experimental factors’ by Hippen *et al.* (12), and can be accessed at https://www.ncbi.nlm.nih.gov/projects/gap/cgi-bin/study.cgi?study_id=phs002262.v2.p1.
